# The Toxic Truth About Carbon Nanotubes in Water Purification: a Perspective View

**DOI:** 10.1186/s11671-018-2589-z

**Published:** 2018-06-18

**Authors:** Rasel Das, Bey Fen Leo, Finbarr Murphy

**Affiliations:** 1 Functional Nano and Micro-Structured Surface, Leibniz-Institute of Surface Modification, Permoserstr. 15, 04318 Leipzig, Germany; 2 Faculty of Medicine, University of Malaya, 50603 Kuala Lumpur, Malaysia; 3 Nanotechnology and Catalysis Research Centre (NANOCAT), University of Malaya, Kuala Lumpur, Malaysia; 4 Kemmy Business School, University of Limerick, Limerick, Ireland

**Keywords:** Carbon nanotube, Water purifications, Physicochemical properties, Risk assessment, Nanosafety

## Abstract

Without nanosafety guidelines, the long-term sustainability of carbon nanotubes (CNTs) for water purifications is questionable. Current risk measurements of CNTs are overshadowed by uncertainties. New risks associated with CNTs are evolving through different waste water purification routes, and there are knowledge gaps in the risk assessment of CNTs based on their physical properties. Although scientific efforts to design risk estimates are evolving, there remains a paucity of knowledge on the unknown health risks of CNTs. The absence of universal CNT safety guidelines is a specific hindrance. In this paper, we close these gaps and suggested several new risk analysis roots and framework extrapolations from CNT-based water purification technologies. We propose a CNT safety clock that will help assess risk appraisal and management. We suggest that this could form the basis of an acceptable CNT safety guideline. We pay particular emphasis on measuring risks based on CNT physico-chemical properties such as diameter, length, aspect ratio, type, charge, hydrophobicity, functionalities and so on which determine CNT behaviour in waste water treatment plants and subsequent release into the environment.

## Background

Gaining access to clean and safe water is a basic human right. Unfortunately, 780 million people throughout the world, especially in developing countries, have no access to fresh water facilities [1]. Carbon nanotubes (CNTs) have emerged as the foremost nanomaterial (NM) for water purification. It can remove almost all three types of pollutants, i.e. organic, inorganic and biological pollutants [2]. This is because of their large surface area, high aspect ratio and greater chemical reactivity along with lower cost and energy. Approximately, 736 metric tons of CNTs were utilized in past few years for energy and environmental applications, a number that continues to increase [3]. Despite the potential for both human and environmental risk, there is no systematic approach to assessing the risks associated with employing CNTs in water purification, a situation that requires urgent attention.

An ample literature study suggests that the frivolous use of CNTs as adsorbents, composites or catalysts, sensors, membranes and engineered NMs is the main reason that 6.0 and 5.5% of CNTs leak from waste water treatment plants (WWTPs) and waste incineration plants, respectively [3]. Alternatively, CNTs could be lost to soil (14.8%) and air (1.4%) from the disposal phase, which might ultimately escape to fresh water bodies. The effects of these environmental CNTs (E-CNTs) are yet to be clear [4]. Our previous research shows how E-CNTs could be transformed [5]. CNTs can be altered to resist biodegradation, increased cellular uptake, reactivity and toxicity to terrestrial, aquatic and aerial flora and fauna. Consequently, societal perceptions may be adversely affected and there may be public pressure to ban CNTs, as they share similar pathological effects to asbestos [6]. All evidence suggests that the public are ignorant of NMs and positively disposed towards the CNT latency effects.

Indeed, the economic sustainability of NMs may depend on appropriate risk weightings applied to the sector [7, 8] or more quantitative approaches [9]. Our literature study concerning CNT safety aspects has suggested knowledge gaps as summarized below:

- There is an absence of universal safety guidelines for CNTs except for the Commonwealth Scientific and Industrial Research Organization (CSIRO) [10].
- While CNT handling as “solid matrices” in the occupational environment or in primary exposure receives priority for risk assessment, extensive knowledge gaps were identified for secondary exposure or environmental pathways.
- Estimating CNT risk was principally based on prior assumptions with less attention paid to significant contributory factors such as CNT’s physicochemical properties in water purification technologies.

**Fig. 1:**
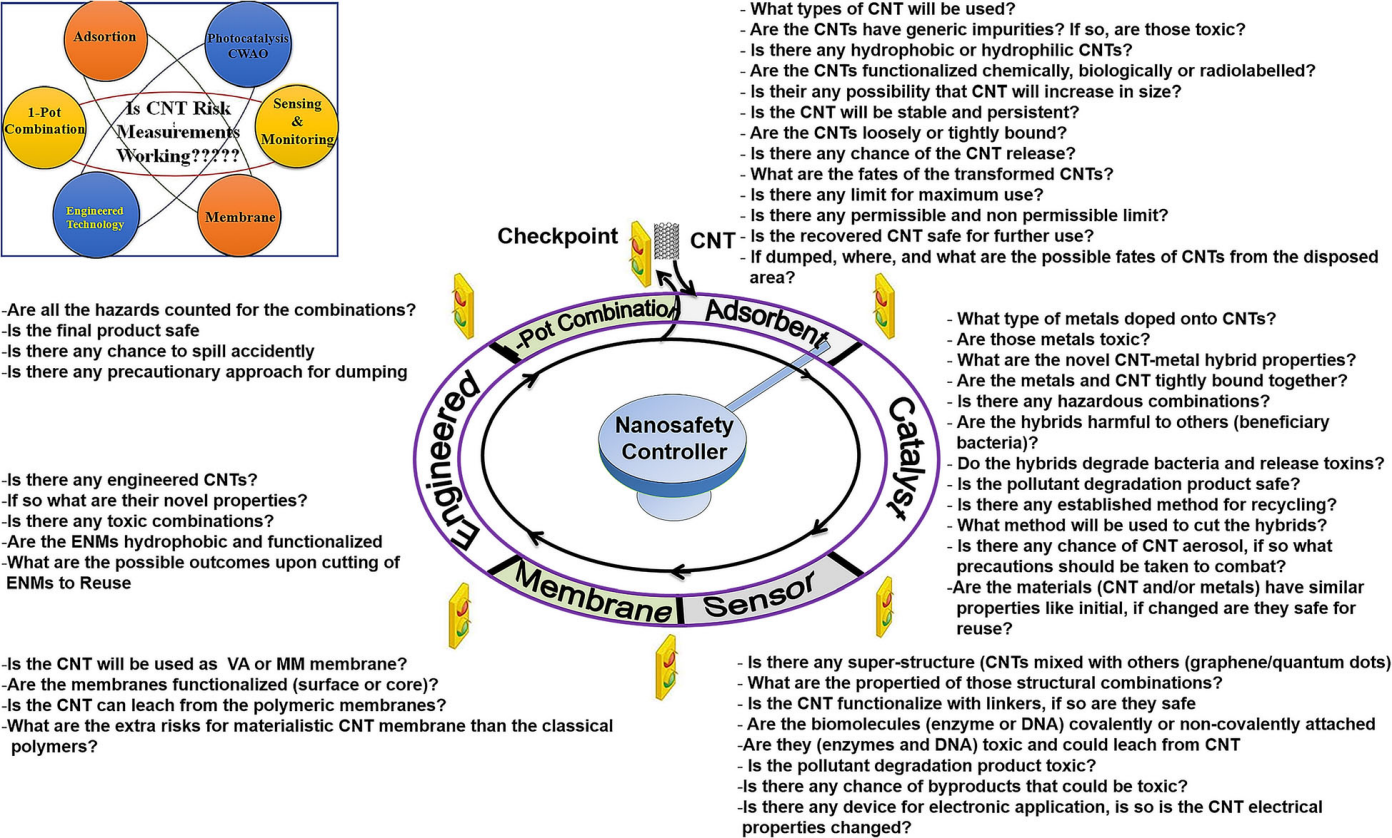
Nanosafety clock. Clockwise rotation pertains to major CNT risk measurements in water purification. These major risks are detailed in subsequent sections of this paper

Although many organizations such as the Environment Protection Agency (EPA), the Organization for Economic Co-operation and Development (OECD), the European Union (EU) and the Centre for Disease Control and Prevention (CDC) have been monitoring the environmental safety implications of NMs, they are still in a “wait and see” approach for E-CNTs. Given the knowledge gaps, here, we postulate several important novel risk assessment and control measurements for E-CNT safety issues as shown in Fig. 1. We emphasize CNT physicochemical properties such as size, shape, diameter, mass, aspect ratio, charge, stability, functionalities controlling aggregation and dispersibility in water, which might affect E-CNT fate and toxicity level. As shown in Fig. 1, specific risk concerns are associated with specific applications of CNTs in water purification. Estimating application-specific CNT risk appraisal and management will help to understand the global scenario and overhaul existing CNT safety guidelines; thus, one can assure nanosafety for CNTs.

## Methods

Carbon nanotubes (CNTs) are fibrous materials formed from honeycomb crystal lattice layers of graphite wrapped into a tube shape either as a single layer or as multiple layers [11]. Precise structural arrangement and order give them a variety of beneficial properties such as ultra-lightweight, high surface tension and high aspect ratio [12]. Single-wall carbon nanotubes (SWCNTs) consist of the cylindrical shape of a single shell of graphene whereas multi-walled carbon nanotubes (MWCNTs) are composed of multiple layers of graphene sheets [13, 14]. Both types of CNTs have been used for direct water desalination and indirect removal of pollutants that complicate the desalination process [15].

It is important to understand that not all CNTs are toxic in which altering shape, size and composition would modify the nanotoxicity of CNTs [16]. CNT with the length of long fibres (> 20 μm) which exceeds the macrophage length cannot be engulfed by macrophage leading to inefficient phagocytosis, and this prevents their clearance from the system, causing harmful effects. Generally, a number of studies have indicated that longer length and larger diameter possess greater toxicity than smaller ones [16]. Furthermore, the length and diameter of CNTs which can be controlled during CNT synthesis are another major factors that determine the life cycle and toxicity. The toxicity of different types of CNTs is summarized in Table 1.

**Table 1:** The toxicity profiles of different types of CNTs in comparison with asbestos (in vivo studies)

Types of CNTs	Diameter/length	Cells types	Result	References
Asbestos	Diameter (0.394 μm ± 1.83 μm) Length (6.22 μm ± 3.22 μm)	Mesothelioma (mice)	Development of extensive inflammatory and proliferative changes. The carcinogenic activity occurred.	[62, 63]
MWCNT (mixture with graphite nanofibres)	Length (5–25 μm) Diameter (10–50 nm)	Lungs (mice) Bronchial epithelial cell (human)	No significant lung inflammation or tissue damage but caused systemic immune function alterations.	[64, 65]
MWCNT	Length (0.5–6.1 μm) Diameter (10–30 nm)	Lungs (rat) Bronchial epithelial cell (human)	Release of pro-inflammatory and pro-fibrotic mediators which could lead to lung fibrosing diseases.	[66–69]
MWCNT (Nanocyl NC 7000)	Length (5–15 nm) Diameter (0.1–10 μm)	Lungs (Wistar rat)	Increase in BALF total cell count, pronounced multifocal granulomatous inflammation and lung fibrotic were the negative effects.	[70]
SWCNT	Length (0.7 μm) Diameter (0.2 μm)	Intratracheal instillation (Wistar rat)	There was no increase in total cell or neutrophil count in BALF. Well-dispersed SWCNT did not induce neutrophil inflammation in the lung.	[71]
SWCNT	Length (0.1–1 μm) Diameter (0.8–1.2 nm)	Lung (mice)	Leads to lung fibrosis effect and acute inflammatory phase reaction were also observed.	[63, 72, 73]
Rigid MWCNT	Length (5.29 μm) Diameter (50 nm)	Mesotheliomas (rat)	Mesothelial injury by thin and thick MWCNTs is responsible for the extent of inflammogenicity and carcinogenicity.	[74, 75]
Long MWCNT	Diameter (> 20 nm)	Mesothelial lining of the chest cavity (mice)	Fibroblast formation proliferation which can lead to pulmonary fibrosis. Formation of lesions known as granulomas and inflammation occurrence were included	[76, 77]

### Life Cycle and Release Dose of CNTs Related to Risk Assessment Studies

**Fig. 2:**
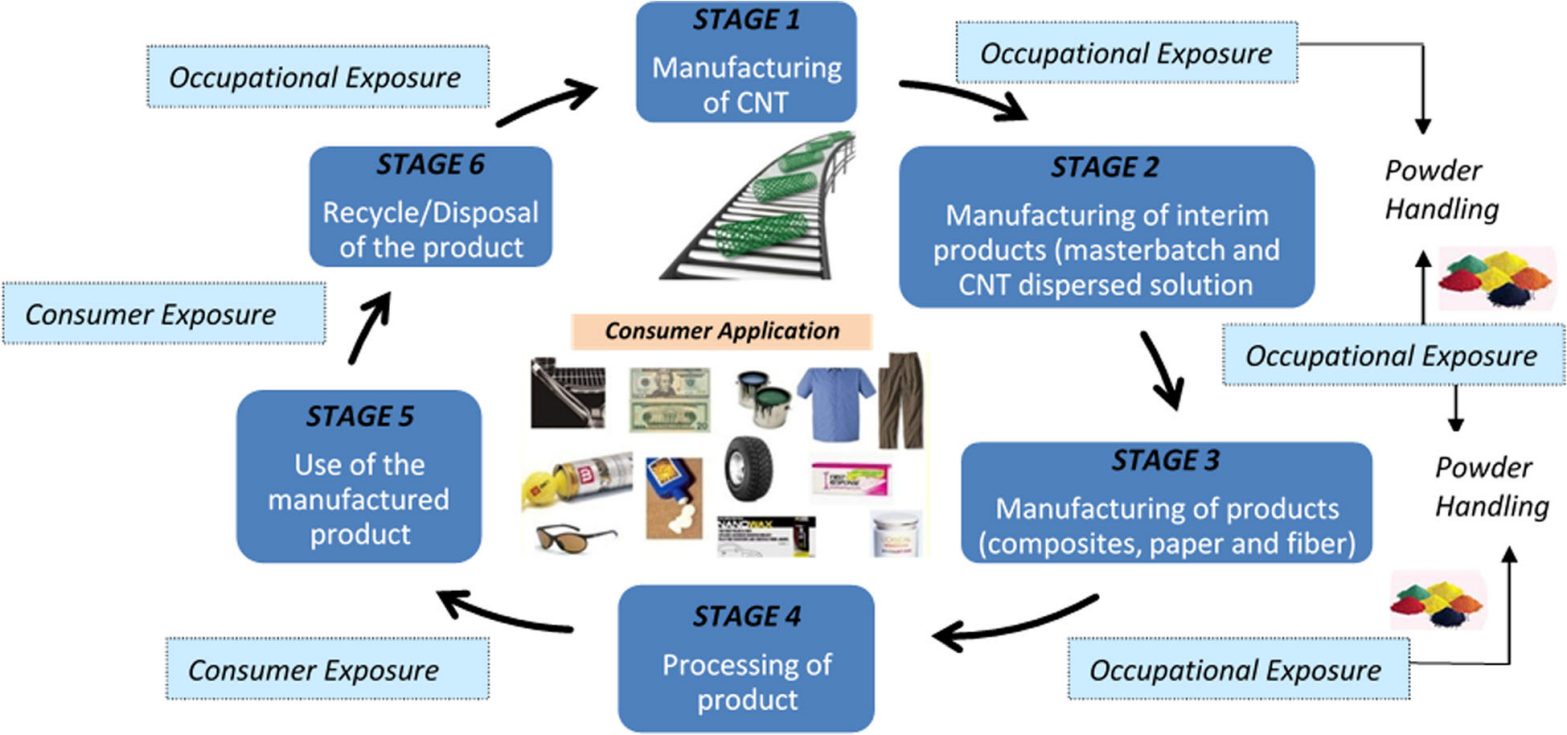
CNT lifecycle. The life cycle of CNT related to risk assessment studies [18, 61]

CNT life cycle can be categorized into six stages as shown in Fig. 2 which relates to their handling quantity and dispersal state [17, 18]. The first stage involves CNT manufacturing which is conducted in an enclosed furnace without oxygen intrusion; thus, the exposure to CNTs is low. Nevertheless, CNT exposure can occur during furnace maintenance and the manual handling of CNTs. The second stage involves the manufacturing of interim products such as masterbatches and CNT-dispersed solutions. Even though the equipment scale and handling quantity in stage 2 are smaller than the production line but agitation in CNT powder process may increase their release rate into the environment. Mechanical abrasion (ware and tare) and physiochemical ageing (corrosion or thermal influence) may cause the release of CNTs. The third stage is the manufacturing of products whereby there will be reduced direct handling of CNTs by utilizing interim CNT-containing products manufactured during the second stage. However, this stage may release some CNTs into the air during solution drying and paint curing. The fourth stage of CNT life cycle is the processing of products in which physical or thermal stress is applied to the composite products whereby CNTs are bound to the base polymer and release of free CNTs from such composite is expected significantly low. The fifth stage is the use of CNT products by consumers, and finally, the six stage is the disposal or recycling of the CNT-based products [17, 18].

Tracking the life cycle of the CNT product may possibly lead to determine in which circumstances a release of CNTs from applications may occur. For instance, CNTs generally embedded in the polymer matrix to enhance mechanical strength, conductivity, etc. will not be released. However, polymer degradation involving photoreaction, hydrolysis, oxidation and thermolysis of the polymer matrix may release CNTs into the environment [19]. The rate of degradation is influenced by the structural features of the polymer as well as external sources such physical, chemical and biological agents that control the processes. Moreover, Wohlleben et al. [20] investigate the life cycle of nanocomposites by comparing released fragments and their subsequent in vivo hazards. The author identifies no significant difference in toxicity for nanocomposite materials in comparison to their traditional counterparts without nanofillers under normal mechanical use (e.g. weathering, normal use phase and sandling). Besides, Wohlleben et al. [21] also analysed the release of CNTs from nanomaterials associated with nano-reinforced tires during their use, either by combined mechanical or chemical stress. The author reports that an on-the-road scenario releases more fragments from stimulated tread wear than the washed to surface water scenario, indicating that only synergistic aging stress induce significant releases.

Research conducted by Girardello et al. [22] on aquatic invertebrate leeches (*Hirudo medicinalis*) analysed acute and chronic immune responses over a short [1, 3, 6, 12] and long (1 to 5 weeks) period of time to exposure to MWCNTs. A massive cellular migration occurred in the exposed leech angiogenesis and fibroplasia. Furthermore, the immunocytochemical characterization using specific markers shows that the monocyte and macrophages (CD45^+^ and CD68^+^) were the most affected cells in these inflammatory processes. These immunocompetent cells were characterized by a sequence of events which begins with the expression of pro-inflammatory cytokines (IL-18) and amyloidogenesis. The author also confirms that aluminium oxide in leech exposure solution was lower than the accepted level for human health in drinking water [22]. Moreover, no metal such as aluminium, cobalt and iron were detected in leech tissues as shown by EDS analysis. This experiment finds that responses in the leeches were caused by the MWCNT and not by the metal oxide presence in the exposure solution [22]. Furthermore, Muller et al. [23] documented that when MWCNTs were introduced into rat tracheas at a dosage of 0.5, 2 and 5 mg per rat, this resulted in inflammatory and fibrotic reactions at all doses after 3 days of single intra-tracheal administration. Research conducted by Xu et al. [24] found that 0.5 ml of MWCNTs (500 μg/ml) inserted five times over 9 days into the lungs of rats results in the presence of MWCNTs in alveolar macrophages and mediastinal lymph nodes.

The aforementioned processes (e.g. CNT synthesis, production of intermediate, further processing, product usage, recycling processes and final disposal) may occur at all stages of the product life cycle [25]. Residual CNTs that remain during the treatment of waste water may form a variety of by-products through a reaction between chemical and some pollutants. Chronic exposure to these chemicals through ingestion of drinking water, inhalation and dermal contact during regular indoor activities may pose cancer and non-cancer risks to humans [26].

Few studies have investigated the fate of CNTs in the environment or their half-life; it is important to consider whether ENMs transform or are transported between different media, and if so, over what timescales. It is becoming well-established that the nature and behaviour of CNTs can alter, sometimes quite radically, depending on the environment they encounter, governed by their physical chemistry, including their surface functional groups, and physical form. The influence on the environment will be controlled by the emergent characteristics of the CNTs and a range of possible mechanisms, including the release of dissolved species, passivation, local depletion of species, or direct CNT uptake by organisms. Besides, the negative effect of CNTs can be minimized by understanding the effects of the physicochemical properties of CNTs on their toxicity. For instance, a research conducted by Wang et al. [27] on decreasing the lung fibrosis potential of MWCNT through pluronic F108 coating finds that the coating was able to confer MWCNT dispersion and reduce profibrogenic effects of these tubes in vitro and in the intact animal lung. The mechanism of this effect has the capacity to prevent lysosomal damage in macrophages and possibly other cell types. The author suggested that PF 108 coating could be applied as a safe design approach for MWCNTs in biomedical fields such as drug delivery and imaging [27].

In summary, to evaluate CNTs’ environmental impact, it is important to accurately characterize them before use and after exposure to different media; the phenomenology at the interface between the nanomaterials and the environment is especially critical for making long-term predictions. There is almost no information available about how ENMs interact with environmental media, and only couple studies have been reported in the field. It is necessary to understand the fate and significance of CNTs released into the environment in order to develop appropriate product designs, safe manufacturing routes, and effective end-of-life disposal strategies.

### Critical Facts for CNTs in Water Purifications

#### Adsorbents

CNTs are a popular adsorbent for water purifications, but some comment on their safety is necessary. Typically, CNTs are required in high volumes for adsorbing water pollutants of extremely high concentrations. Thus, it is necessary to see what types of CNTs are deployed and how much is being used. Different CNT individuals might have different physicochemical properties which should be accessed. Over 50,000 different types of CNTs are available in the market [28] with different lengths, shapes, charges and so on that exemplify the complexity of the material in the environment. On the other hand, pristine CNTs are themselves problematic because of their generic impurities [29] such as metals and carbonaceous agents that pose nanosafety issues. As a corollary, scientists have purified and functionalized CNTs using different approaches [30, 31], but a recent study demonstrates that such CNTs increase the metal uptake and toxicity levels on living cells [32].

Adsorbing water pollutants changes the CNT characteristics such as pore size and volume, surface charge or energy, stability, hydrophobicity and functionalities [33]. Firstly, the adsorption of various organic water pollutants such as humic acid and tannic acid (TA) alters CNT properties and increases its stability in the environment. Hyung et al. found stable CNT with adsorbed organic matters in the Suwannee River water [34], consistent with the study of stable fullerenes in the Sahan River, Ukraine [35]. Transmission electron microscopy (TEM) images suggested that the CNTs were thick in sizes upon TA adsorption and led to the separation of individual CNT from the bundle [36]. Similar phenomena can also be found for surfactant adsorption onto the CNTs, which changes the nanotube’s dispersibility in water [37]. These studies postulate that stable CNTs may be transported and subsequently deposited after their release from WWTP into aqueous environments, thus leading to the potential uptake of E-CNTs by living cells. Secondly, inorganic metals such as Fe, Cd, Ni, As and Hg adsorbed onto the CNTs could have greater reactivity and toxicity within the particle. Studies found that CNTs with metal ions such as Fe and Ni are more toxic to living cells [38]. Moreover, biological adsorbents, especially microbes, have the potential to change CNT surface properties in WWTPs. For instance, some bacterial intracellular enzymes catalyse the formation of hydroxyl radical (^**•**^OH) or H_2_O_2_ through redox reactions that produce carboxylated (C)-CNTs [39]. This converts hydrophobic pristine CNTs to hydrophilic ones, affecting their aggregation and making their handling extremely difficult, and the tubes would be difficult to hold in the WWTP. Some enzymes have degraded C-CNTs [39, 40] and transformed the short CNT fragments to facilitate subsequent transportation in the environment. Therefore, the pollutants (e.g. organic, inorganic and biological) should be removed in such a way that the CNT properties would not be changed. One should check whether there covered CNT after adsorption has been cut, ground, sheered and ripped or not. On that basis, one can predict the suitability of CNTs to reuse for pollutant adsorptions.

### Catalysts for Advanced Oxidation Processes

Measuring CNT risks as catalyst composites is possible in multiple ways. First, alloying CNTs using metals such as Ti, Cr, Mn, Fe, Co, Ni, Cu, Zn, Mo, Rh, Pd, Ag, Cd, Pt, Au, Hg and their oxides through physical and/or chemical adsorptions is not stable; there is a chance of releasing significant amount of metal particles into the environmental milieu. Secondly, each doped metal has its own specific properties that might influence parental CNT’s properties and ultimately the overall behaviour of the composite. For example, Fe is popular to magnetize the CNT catalyst for ease of recycling, which could generate hydroxyl radicals that affect cell viability [41]. These might impact nanosafety risk assessment strategies, and one should count the final composite’s biocompatibility, health risks and toxicity issues before developing a safety guideline. Thirdly, disinfection of microbes using CNT’s composite is important. CNT-Ag-TiO_2_ has shown direct antimicrobial effects and is popularly used for rupturing bacterial cell walls [42]. However, such treatment could be lethal, since a few bacteria especially Cyanobacteria could be responsible for releasing more toxic compounds, i.e. microcystins, while decontaminating through the CNTs [2]. Fourthly, photodegradation and catalytic wet air oxidation (CWAO) of persistent organic pollutants using CNT-metal catalysts have produced various degradation products and/or their intermediates which could be more toxic than their parent compounds and harmful to health [43]. Therefore, before assuming that CNT-metal composites are completely safe to use as a photocatalyst and catalytic wet-air oxidant, one should also keep in mind the degraded product’s reactivity, toxicity and fate in the environment. Finally, scientists need to isolate parental CNTs from doped metal for recycling. Although dry or wet cutting techniques are available for cutting and/or grinding of CNT composites [44], there is a significant chance of creating aerosols of free short CNT/metal fragments. Surface water and lands will be the ultimate destinations of any atmospheric release of CNTs and should be treated with caution. Therefore, handling CNT-metal composites in liquid media or instating extraction ventilation while processing will be helpful.

### CNT Application in Sensor Manufacturing

Application of CNTs as an electrode for biosensors is comparably safe to use. There is little chance of direct water contact with the CNT electrode. However, a few risk measurements can be followed. Firstly, 1D CNTs are often combined with 2D NMs, especially graphene for high electroconductivity and mechanical flexibility. Such superstructures have different physicochemical properties [45] and pose different environmental hazards which should be measured with caution. Secondly, poly (diallyldimethylammonium chloride) (PDDA)-functionalized CNTs are very common in electrochemical biosensors. CNTs-PDDA is harmful since the polymer has influenced cell viability and haemolysis [46]. Finally, biomolecules such as deoxyribonucleic acid (DNA), aptamers, enzymes and proteins have been widely immobilized onto CNTs for sensing organic, inorganic and biological water pollutants. The preferable immobilization method of these biomolecules is physical adsorption rather than covalent modifications in order to maintain the CNT’s integrity and biomolecule’s conformations that lead to high electrical conductivity. However, such system is not stable and durable since biomolecules leached from the system are often toxic to humans. Therefore, the quality of a biosensor and its risk quantifications are completely dependent on strategies taken to produce the final product.

### Utilization of CNTs in Membrane Production

CNTs are popular as separate membrane itself called vertically aligned (VA)-CNT membrane. In contrast, mixed matrix (MM)-CNT membrane could be generated by doping CNTs into the existing polymeric membranes such as reverse osmosis (RO), nanofiltration (NF) and ultrafiltration (UF) for the enhanced separation process. Therefore, researchers often classify CNT membrane as RO, NF, UF and nano-enhanced membranes [47]. This is not acceptable—at least from a nanosafety viewpoint since CNT membrane is different from the RO, NF and UF membranes. According to the International Union of Pure and Applied Chemistry (IUPAC) and the International Organization for Standardization (ISO), a membrane could only be classified on the basis of the size of the water pollutant that they reject [48, 49]. While RO and NF membranes purify water at diffusion, UF membrane retains suspended water particles. In contrast, a CNT membrane holds both dissolved ions and suspended solids and has also been used for gas separation [50]. While organic polymers are the building blocks of RO, NF and UF; CNT is a carbon allotrope. Compared to conventional membranes, CNT membranes are often functionalized with other nanoparticles such as TiO_2_, Ag and Fe_3_O_4_ which might have different physicochemical properties. As a result, conventional risk assessments for RO, NF and UF cannot be applied to CNT membrane. One should consider both the conventional and newly emerging risks associated with CNT membrane technology. Therefore, CNT safety guidelines as a membrane process should be based on materialistic and applied viewpoints not merely based on the inconsistent use of terminology given by scientists. The classification of CNT membrane should be critically reviewed in order to regulate them in the light of risk estimation and regulations because it is not possible to enact laws without clear definitions of the technology.

### Engineered Nanomaterials

Engineered CNTs are making remarkable promises in water purifications [51]. It has been calculated that about 1100–29,200 metric tons/year of engineered nanomaterials (ENMs) are emitted from WWTP as effluents worldwide [52]. Hours and days later, such ENMs are settling as larger aggregations in natural water resources. Therefore, the successful use of ENMs requires the implementation of safety guidelines [53] on the basis of its novel properties such as shape, size, charge, agglomeration and so on. The unusual reactivity of ENMs is because of their surface and quantum effects with different optoelectronics and mechanical properties [54]. Such properties need to be verified because of their various toxicological outcomes. The fate of engineered CNTs depends on its interfacial properties, such as adsorption, reactivity, adhesion, cohesion and wettability, and also regulated by water chemistry such as pH, pollutant mixtures and so on [54]. Engineered CNTs with appropriate functionalities act as a point of attachments where different natural water constituents can anchor. Such modification would facilitate the separation of CNTs from the bundle, and individual CNTs will leak out from WWTP. Therefore, contaminated water effluents could be found in water treated by CNTs. Because of the material complexity, it is often difficult to measure the toxicity of CNTs. Scientists use assumptions such as “One Size Fit All” for measuring toxicity phenomena of these complex novel materials. There is a knowledge gap and a paucity of scientific data. Some thought is required to validate and check the toxicity levels of each ENM precisely. Besides wet lab works, we can anticipate using some computational tools such as quantitative structure-activity relationship (QSAR) models for classifying the ENMs with consensus physicochemical properties. This will help stake holders understand the overall risk hot-spots and enable them to choose which combination would be safe to use. Scientists can also bracket threshold limits for each ENM to be used in WWTPs.

### One-Pot Combined Technology

Scientists often prefer to develop “One-Pot” technology where different water purification technologies will be integrated to tackle multiple water pollutants in real-time [5]. Tracking of such combinations in terms of nanosafety can be a difficult job. To our knowledge, no toxicity test of such hybrid technology has yet been done, so one might need to test for any environmental harm. Obviously, the risk assessment for each separate technology should be concerned with others so that one can implement the controls without further assessment. The total risk of “One-Pot” combined water purification technology can be calculated as follows:

$$ \mathrm{Total}\ \mathrm{risk}\mathrm{s}=\mathrm{level}\ \mathrm{of}\ \mathrm{risk}\ \mathrm{appraisal}\ \mathrm{of}\ \mathrm{combined}\ \mathrm{technologies}\times \mathrm{severity}\ \mathrm{of}\ \mathrm{their}\ \mathrm{hazards} $$

### Occupational Exposure Risks of CNTs

An increase in the number and production volume of products containing engineered nanomaterials (ENMs), however, will lead to a larger release in the environment during manufacture, use, washing or disposal of the products [55]. At a simple level, nanotechnology would seem like a safe industry since very few problems have been reported to date. However, the most adverse effects of these ENMs may become apparent over time and provide liabilities similar to asbestos-containing products due to their pervasive use in daily life. ENMs as potential occupational and environmental hazards may raise health and safety concerns [56]. As reported by NIOSH, seven workers developed hypoxaemia and severe lung disease after working with a chemical paste comprising a mixture of undefined nanoparticles (NPs). In terms of occupational health exposure risk, data has emerged providing evidence that a worker died due to respiratory distress syndrome while spraying nickel NPs onto bushes for turbine bearings using a metal arc process. Unfortunately, the nanotechnology industry has remained largely silent on the use of ENMs, and government regulators have not introduced strict guidelines. For this reason, there is a need to assess the toxicity of ENMs and understand their possible benefit or adverse effects on human health.

The effect of CNTs appears to be correlated with their method of administration or exposure [16]. The updated available standard is prescribed for asbestos whereby the permissible exposure limit (PEL) is 0.1 fiber per cubic centimetre of air over an 8-h time-weighted average (TWA) with excursion limit (EL) of 1.0 asbestos fibers per cubic centimetre over 30-min period. The employer must ensure that no one is exposed above this limit. Monitoring workplace or work activity to detect asbestos exposure is at or above PEL or EL for a worker who is at risk of exposure is crucial [43].

A number of studies have reported that the exposure of CNTs to the respiratory system could lead to asthma, bronchitis, emphysema and lung cancer. It is important to note that some factories are dustier possibly due to the lack of industrial hygiene standards [4]. Working with pulverized CNTs or mixtures that contain fine CNT particles could pose a risk of inhalation. Many experimental studies conducted on inhalation exposure have contributed to the assessment of the effects of CNTs on respiratory tract and identification of exposure limits. Prolonged occupational exposure to airborne CNT matter could lead to severe lesions in the lungs as documented in animal studies [4].

## Results and Discussion

The functionalized nonpolar interior home of CNT provides a strong attraction to polar water molecules and rejects salt and pollutant. This, accompanied by low energy consumption, antifouling as well as self-cleaning function has made CNT membranes an extraordinary alternative to conventional water treatment technology [47]. Pristine CNTs often consist of various metal catalysts, ash and a carbonaceous agent which act as additional adsorbent site of CNTs for multiple water pollutant. The impurities are one of the factors used to identify nanotubes’ pore diameter, morphology and ability to influence or inhibit adsorption behaviours [57]. Impurity reduction and removal without affecting the original nanotube integrity is one the major challenges in CNT-based water purification applications [5]. Several methods have been applied to get intact CNTs such as filtration, high-temperature annealing and repetitive centrifugation, but the methods are still unable to completely remove the CNTs [5, 58, 59].

Besides CNT purification, manipulation of CNT solubility in the water system is one of the major impeding factors in water purification technology. As an example, pristine CNTs are insoluble in water due to their hydrophobic graphite sheet [5]. In order to counter this shortcoming, a covalent modification has been applied whereby hydrophilic substituent is introduced using wet chemical treatment. Another method is non-covalent modification which complements the surfactant wrapping that is widely used to increase CNT solubility in water or different aqueous media [60]. CNT contamination in the environment could occur when nanotubes leaked from the water purification column during operation and directly flows into surrounding water resources. These CNTs have a high chance to react with various biomolecules present in the water system which possibly could generate toxic effects to the surrounding aquatic environment [5]. Even though CNTs could offer efficient water purification technologies, the potential environment effects need to be critically analysed in order to estimate risk and develop safety guidelines in the use of CNT materials in water treatment systems.

## Conclusions

Ensuring clean and safe water facilities, preserving our environment and avoiding societal nanophobia are some of the challenges faced by scientists and those involved in the use of nanomaterials. We must ensure the connectivity of each step in the handling, use, disposal and fate of CNTs in water purification technologies. At present, there is a paucity of methods and criteria for accurately measuring CNT risks and hazards. It is apparent that there is a need for solid regulatory frameworks that address and specifically manage the potential risks of nanotechnology. This regulatory framework should address the challenges faced in identifying and characterizing the nanomaterial form and its impact on human health and the environment. Our case-by-case, in-depth risk assessment procedures based on the nanomaterial’s structure-property relationships will help in understanding CNT behaviour in WWTPs and their subsequent release into the environment. With the help of these relationships, a universal safety guideline can be developed to accurately address risk estimates of CNTs in future water purification applications.

## Abbreviations

CDC: Centre for Disease Control and Prevention
CNTs: Carbon nanotubes
CSIRO: Commonwealth Scientific and Industrial Research Organization
CWAO: Catalytic wet air oxidation
E-CNTs: Environmental CNTs
EPA: Environment Protection Agency
EU: European Union
IUPAC: International Union of Pure and Applied Chemistry
MM: Mixed matrix
MWCNTs: Multi-walled carbon nanotubes
NM: Nanomaterial
OECD: Organization for Economic Co-operation and Development
PEL: Permissible exposure limit
QSAR: Quantitative structure-activity relationship
SWCNTs: Single-wall carbon nanotubes
TA: Tannic acid
TEM: Transmission electron microscopy
TWA: Time-weighted average
WWTP: Waste water treatment plant
